# Effectiveness of web-based education in addition to basic life support learning activities: A cluster randomised controlled trial

**DOI:** 10.1371/journal.pone.0219341

**Published:** 2019-07-11

**Authors:** Helene Bylow, Thomas Karlsson, Margret Lepp, Andreas Claesson, Jonny Lindqvist, Johan Herlitz

**Affiliations:** 1 Department of Molecular and Clinical Medicine, Institute of Medicine, Sahlgrenska Academy, University of Gothenburg, Gothenburg, Sweden; 2 Health Metrics Unit, Institute of Medicine, Sahlgrenska Academy, University of Gothenburg, Gothenburg, Sweden; 3 Institute of Health and Care Sciences, Sahlgrenska Academy, University of Gothenburg, Gothenburg, Sweden; 4 Østfold University College, Halden, Norway; 5 School of Nursing and Midwifery, Griffith University, Brisbane, Australia; 6 Department of Medicine, Centre for Resuscitation Science, Karolinska Institute, Stockholm, Sweden; 7 Centre of Registers Västra Götaland, Gothenburg, Sweden; 8 Prehospen-Centre of Prehospital Research; Faculty of Caring Science, Work Life and Social Welfare; University of Borås, Borås, Sweden; Murray State University, UNITED STATES

## Abstract

**Background:**

Effective education in basic life support (BLS) may improve the early initiation of high-quality cardiopulmonary resuscitation and automated external defibrillation (CPR-AED).

**Aim:**

To compare the learning outcome in terms of practical skills and knowledge of BLS after participating in learning activities related to BLS, with and without web-based education in cardiovascular diseases (CVD).

**Methods:**

Laymen (n = 2,623) were cluster randomised to either BLS education or to web-based education in CVD before BLS training. The participants were assessed by a questionnaire for theoretical knowledge and then by a simulated scenario for practical skills. The total score for practical skills in BLS six months after training was the primary outcome. The total score for practical skills directly after training, separate variables and self-assessed knowledge, confidence and willingness, directly and six months after training, were the secondary outcomes.

**Results:**

BLS with web-based education was more effective than BLS without web-based education and obtained a statistically significant higher total score for practical skills at six months (mean 58.8, SD 5.0 vs mean 58.0, SD 5.0; p = 0.03) and directly after training (mean 59.6, SD 4.8 vs mean 58.7, SD 4.9; p = 0.004).

**Conclusion:**

A web-based education in CVD in addition to BLS training enhanced the learning outcome with a statistically significant higher total score for performed practical skills in BLS as compared to BLS training alone. However, in terms of the outcomes, the differences were minor, and the clinical relevance of our findings has a limited practical impact.

## Introduction

Globally, cardiovascular disease (CVD) is the leading cause of death [1]. Education in basic life support (BLS) for laymen (non-healthcare professionals) includes a theoretical knowledge of out-of-hospital cardiac arrest (OHCA) and first and foremost training in the practical skills of cardiopulmonary resuscitation (CPR) and the use of an automated external defibrillator (AED). The early initiation of high-quality CPR-AED increases survival from OHCA dramatically [2–6]. There is an association between the increase in the proportion of people trained in CPR and the increase in the proportion of patients who receive CPR before the arrival of the emergency medical service (EMS) [3].

Learning activities for BLS for the general public in Sweden include a limited theoretical knowledge of CVD for the benefit of practical skills training [6]. The early recognition of an acute life-threatening situation, calling the EMS and starting treatment are essential. Awareness of how to detect a victim with stroke, acute myocardial infarction (AMI, i.e. heart attack) or sudden cardiac arrest (SCA) and how to treat him/her instantly is important for survival [7–10]. Statistical updates on heart disease and stroke emphasise healthy lifestyle factors for cardiovascular health [1]. The Swedish National Stroke Campaign (strokekampanjen.se, the Swedish Association of Local Authorities and Regions) increased the public awareness of symptoms, how to identify and call 112 when a victim suffered a stroke, both directly after the campaign and nine months after the campaign. However, this knowledge decreased after 21 months [9, 10]. The web-based education named Help-Brain-Heart (hjalphjarnahjarta.se, Swedish Resuscitation Council) on CVD and SCA also improved theoretical knowledge about CVD. However, when compared with traditional BLS, there was no significant difference regarding practical skills in CPR six months after training for thirteen-year-old students [11].

Learning can be effective and constructed in different learning activities. For example traditional learning in the classroom (face-to-face) and digital learning (e-learning) outside the classroom (flipped classroom) [12]. Blended learning with e-learning prior the course combined with instructor-led training in the classroom have been reported to be as effective as traditional training for both basic and advanced life support courses [6, 13–17]. Both the American Heart Association (AHA, www.heart.org) and the European Resuscitation Council (ERC, www.erc.edu) and the Swedish Resuscitation Council (www.hlr.nu) advocate e-learning prior to courses. According to Kolb’s experiential learning theory, experience is a key for learning in a four-stage learning cycle: (1) concrete experience, (2) reflective observation of the new experience, (3) abstract conceptualisation and (4) active experimentation. Education of adult laymen in a community with general life experiences and some awareness on cardiac arrest deal with both new learning and re-learning. CPR training activate practical skills in a concrete experience. Together with reflection on theoretical knowledge in the form of for example digital technology, abstract thinking and testing the skills in training may improve effective learning according to the theory [18]. Therefore, Kolb’s learning theory can add perspectives and gain knowledge of the participants learning in BLS.

Little is known about the efficacy and learning outcome of digital learning in addition to BLS learning activities relating to practical skills and knowledge among laymen in Sweden. There is a knowledge gap of results from large randomised controlled trials (RCT), with a population from workplaces in the society including laymen.

The aim of this trial was to compare the learning outcome in terms of practical skills and knowledge of BLS and CVD after participating in learning activities in BLS, with and without a web-based education in CVD.

The primary hypothesis was that the BLS training plus the web-based education (BLS+WEB) group would achieve a higher total score for practical skills in BLS compared with the BLS group six months after training (retention test) and directly after training (post-test), as secondary hypothesis. Other secondary hypotheses included that the BLS+WEB group would achieve a higher score for the quality of practical skills, theoretical and self-assessed knowledge and confidence and willingness to start CPR-AED in a real-life OHCA situation directly and six months after training as compared with the BLS group.

## Methods

### Study design

This study was designed as a parallel cluster randomised controlled trial (RCT) with a study population recruited from a BLS educational intervention project directed at laymen working at non-healthcare workplaces in Sweden in 2014–2016. The BLS education was based on the 2010 ERC guidelines [19, 20] and both CPR and AED were included. The education was facilitated in total, by sixteen updated independent instructors and co-ordinators at the workplaces. The design and instruments were tested in 2013 in a pilot study [21]. This study was registered at ClinicalTrials.gov (ID: NCT03618888). The CONSORT statement (www.consort-statement.org) was used for the study design and to report a cluster randomised controlled trial (S1 Checklist) [22].

### Study population

The study population consisted of the public in the community. Selection and recruitment were made through the BLS educational intervention project. The inclusion criteria were laymen, eighteen years of age or older, with no previous BLS training (in CPR 35.5% and in AED 79.6%) or no BLS training within the past five years (in CPR 64.5% and in AED 20.4%). The participants represented 84 workplaces and three counties in the south of Sweden. The participants gave their informed consent (S1 Text). The exclusion criteria were healthcare professionals, if they had participated in BLS education within the past five years or if they were unable to perform the six-month practical test. The study enrolled 3,011 participants, cluster-randomised 2,623 individuals and 2,529 were eligible for analysis.

### Randomisation

The participants were cluster randomised to either BLS education or to BLS+WEB education. An online service (www.randomizer.org) was used to generate a list of numbers in blocks of 25 participants in 112 clusters. An independent coordinator who worked in the central municipal house delivered the generated interventions. The study participants and the instructors were aware of an educational intervention but not of the randomisation, the different exact training alternatives or the objective. The investigator was blinded in accordance with a PROBE (Prospective Randomised Open Blinded End-Point Evaluation) design [23].

### Intervention

**Fig 1:**
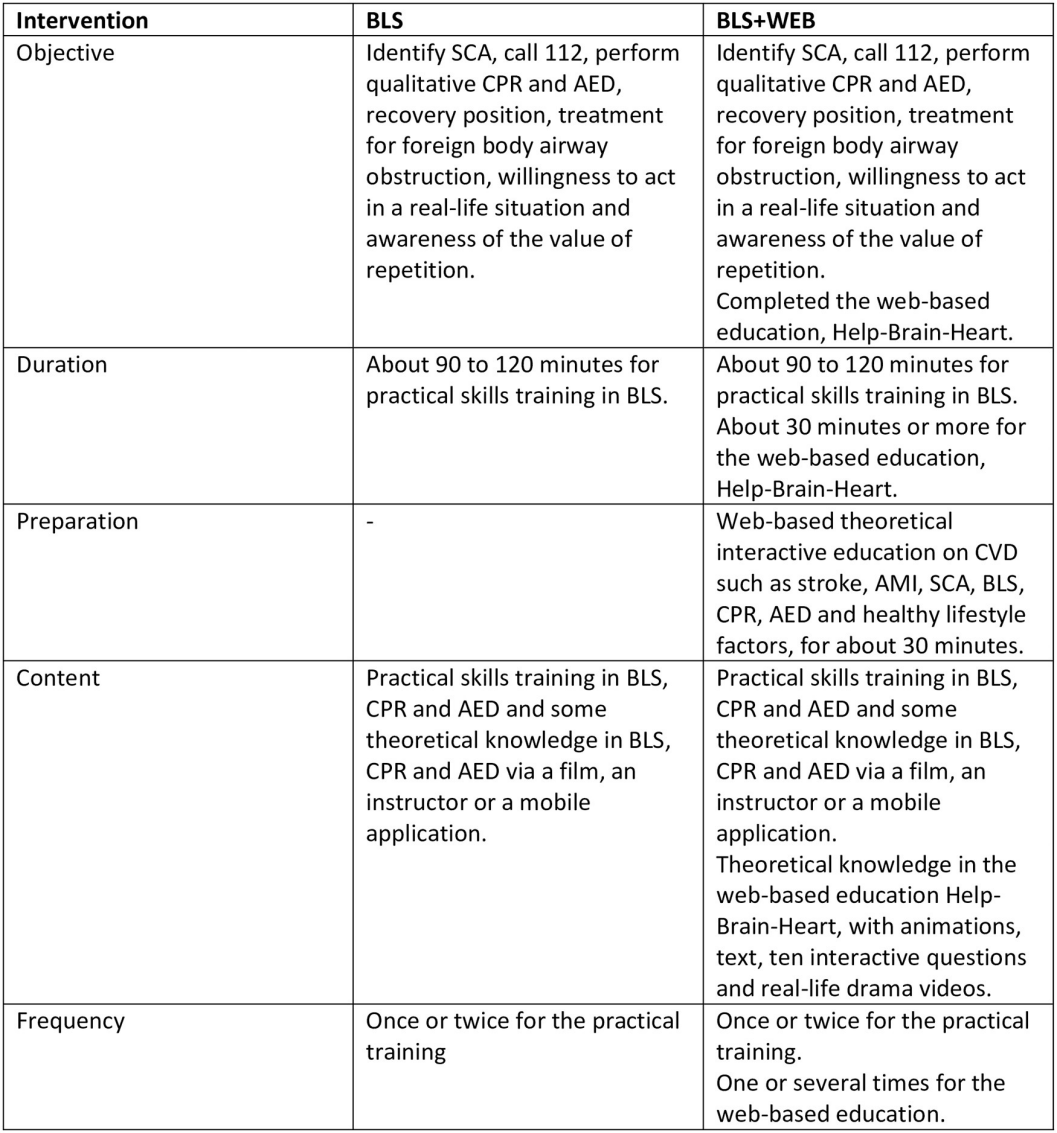
Description of the BLS intervention. Description of the educational intervention in the cluster randomised controlled trial, comparing learning outcome after training in basic life support (BLS) with training in basic life support including a preparatory web-based education on cardiovascular disease (BLS+WEB). Abbreviations: BLS, basic life support; BLS+WEB, basic life support plus a web-based education; SCA, sudden cardiac arrest; CPR, cardiopulmonary resuscitation; AED, automated external defibrillation; CVD, cardiovascular disease; AMI, acute myocardial infarction.

The intervention for both groups was based on learning activities in BLS. The control group constitute participants who received BLS training without the web-based education. The intervention group constitute participants who received BLS training with the web-based education (Fig 1).

### Education with BLS

The BLS education including practical skills training was included in both groups with equal training manikins and cardboard training AEDs (MiniAnne, Laerdal Medical, Stavanger, Norway) for practice while watching an instruction film. The training was instructor-directed or self-directed. The instructor-directed training took place in groups of 12–25 participants (one or two instructors/group) and lasted for about 90–120 minutes. The film lasted for 60 minutes and contained an OHCA situation and practical instructions on how to perform CPR and use an AED. The self-directed training contained the same 60-minute film or a mobile application which lasted for 30 minutes with the same instructions but without the OHCA situation. The self-directed participants were able to attend the instructions and practical training as many times as they wanted for about two weeks. The BLS training alone constituted the control group.

### Education with BLS+WEB

The BLS+WEB group (intervention) completed a web-based interactive theoretical education named Help-Brain-Heart (www.hjalphjarnahjarta.se, SRC) that lasted for about 30 minutes, before the BLS training. The web-based education included theoretical knowledge of the symptoms of CVD, stroke, AMI, OHCA and when to call 112, how to perform CPR and use an AED. It also contained information on healthy lifestyle factors and animations and real-life drama videos on stroke and OHCA. The education was interactive and ten multiple-choice questions gave feedback both textually and with a voice prompt for both correct and incorrect answers. Some of the questions included different features such as drag and drop. In order to proceed, the correct answer was required. After completing the web-based education, a certificate was generated. The participants in the BLS+WEB group were able to attend the web-based education as many times as they wanted.

### Assessment and data collection

Data was collected from assessment of the participants adherence to the BLS algorithm and practical skills and of theoretical knowledge by questionnaires directly after intervention (post-test) and six months after intervention (retention test).

Theoretical knowledge was assessed by questionnaires directly after training and six months later (S2 Text). The questionnaire, in total 38 questions on a paper, both open and closed, was developed from previous studies [7–11, 24, 25] and based on national and international guidelines [19]. The questionnaire asked for background factors as age, gender, weight, height, previous CPR training, level of education, occupation and previous experience from a real-life stroke, AMI or SCA situation. It also asked for theoretical knowledge of call to 112, symptoms of stroke, AMI and SCA, healthy lifestyle factors, and self-assessed theoretical and practical knowledge, confidence and willingness to act in real-life cardiac arrest situation on a relative and an unknown person. The total score was calculated for each question with correct answer and an individual score was calculated for each question answered with a yes.

The practical skills were assessed directly or within one day after the training and six months after the training. The equipment that was used was a Laerdal Resusci Anne manikin and a Heart Start 1 Trainer. The PC SkillReporting system 2.4.1 (Laerdal Medical, Stavanger, Norway) measured quantitative data on performed CPR and the time for a uniform data entry. Calibration of the computer program that was connected to the training manikin was made automatically by the program at each start-up and by the assessor who manually checked the equipment before the assessment. An extra identical training manikin and training AED was prepared in case of technical problems.

The Cardiff Test of basic life support and automated external defibrillation (Cardiff Test) [26, 27], adapted to the 2010 ERC guidelines [19] and modified from previous studies [11, 28–31], was used to calculate the total score for practical skills (S3 Text). To control the observed skills, a Sony HD video camera recorded the practical assessment. The environment was the same for all participants in a private room, with a dressed manikin on the floor. The total time for the assessment was three minutes to identify the victim and perform CPR and about two minutes to use the AED. The participants were only aware of a follow-up meeting and the assessment started after brief information about a simulated video-recorded scenario. Permission and a signed consent form were required. A simulated scenario situated as though it was at the workplace with a collapsed colleague was then described by the assessor (HB) and the participant was told to act as if it was a real-life situation. Three minutes from the start, the assessor placed the AED next to the victim and, after one shock and the resumption of CPR, the scenario was terminated.

The variables for measuring theoretical knowledge in the questionnaire were self-assessed theoretical and practical knowledge of performing compressions and ventilations and using an AED, self-assessed confidence and willingness to start CPR on a relative or an unknown person who had an OHCA, symptoms of stroke and AMI, knowledge of first action in the event of stroke, AMI or OHCA and healthy lifestyle factors.

The variables for measuring adherence to the BLS algorithm used for the total Cardiff Test score (minimum 19 points and maximum 70 points) were assessed with direct observation and the Skill Reporting system. The variables were check for responsiveness and breathing, call for help, ask for an AED, start and ratio of CPR, hand placement, depth and rate for compressions, volume and rate for ventilations, attach electrode pads, safety check and use of the AED and resume CPR after shock. Separate variables measured with the Skill Reporting system and related to CPR quality skills were compressions, ventilations, time to start of compressions and time to shock.

### Outcomes

The primary outcome was the total score, calculated with the Cardiff Test, for adherence to the BLS algorithm, six months after training. The secondary outcomes included the total score directly after training and the quality of separate variables related to CPR and AED, theoretical knowledge of first action in the event of stroke or SCA, symptoms of stroke and AMI, healthy lifestyle factors and self-assessed knowledge and confidence and willingness to act in a real-life OHCA situation, directly and six months after training.

### Statistical analysis

In the analyses, all the available data were included. The data are presented as crude numbers and proportions (percent) or as crude means with standard deviation (SD) and medians with 25^th^, 75^th^ percentiles.

To detect a two-point difference in the mean of the total score for the modified Cardiff test at the retention test after 6 months (primary outcome), with an assumed standard deviation of 5 points, a significance level of 0.05 (two-sided test) and a power of 95%, an effective sample size of 163 participants in each of the two training groups was needed. The intraclass correlation coefficient was 0.062. Based on an average cluster size of 23.4 the design effect caused by the cluster randomization was calculated to be 2.40. In our two training groups 1268 and 1212, respectively, performed the retention test, which corresponds to an effective sample size of 529 and 506, respectively, i.e. well above the 163 needed to reach a power of 95%.

Mixed linear regression models were applied for comparisons of the total score and other continuous measurements to account for a potential cluster effect in the training groups. Generalized estimation equations (GEE) analysis with logit link function was applied for comparisons of proportions. Fisher’s exact test (i.e. without accounting for clustering) was performed for comparisons when proportions were very small (<1.0%).

All comparisons between the training groups were adjusted for the possible confounding influence of age, educational level and occupation at training because of the imbalance between the two groups regarding some of the participants’ characteristics. The analyses accounted for the possible effect of additional interventions (i.e. instructor-led training, instructions from a video film, reflective questions and the feedback device on compression depth).

All tests were two-sided and p-values below 0.05 were considered statistically significant. SAS for Windows, version 9.4, was used for all the performed analyses.

### Ethics

Ethical approval was granted (23 March 2014/134-14) by the Regional Ethical Review Board in Gothenburg.

## Results

**Fig 2:**
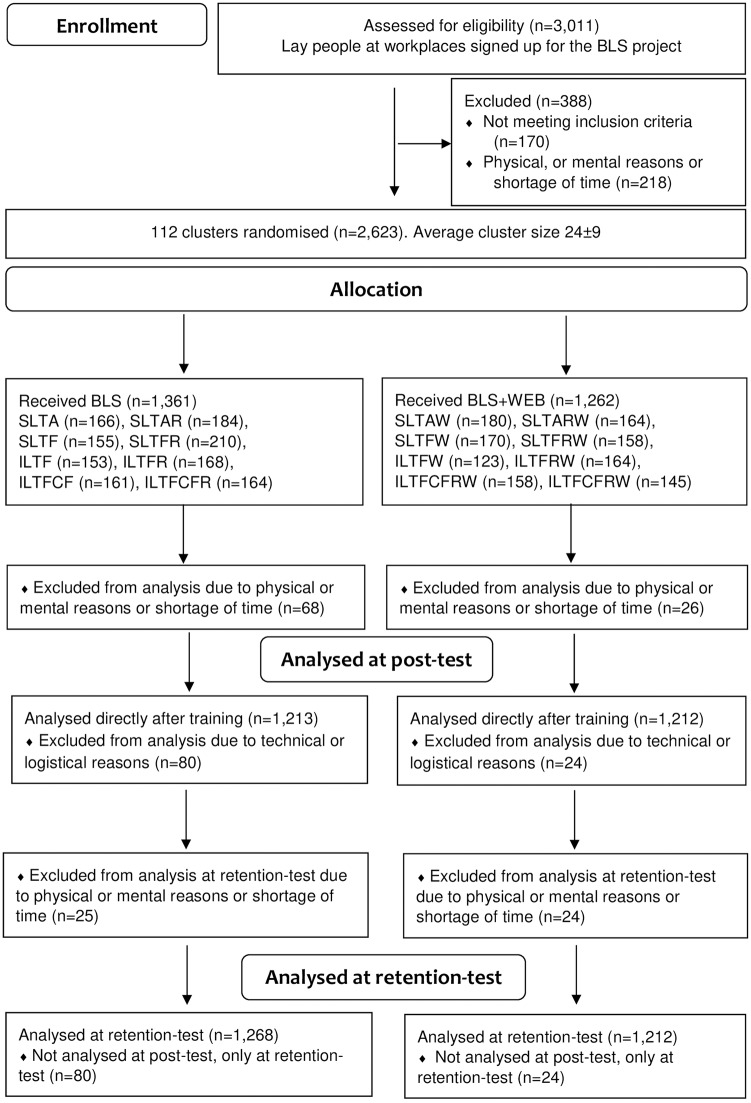
Flow-chart. Flow chart according to CONSORT (www.consort-statement.org), to compare the effectiveness of basic life support (BLS) education with preparatory web-based education in cardiovascular disease (CVD) in addition to BLS training (BLS+WEB). The participants were randomised, educated in BLS and assessed for their total score in practical skills of BLS. The post-test was performed directly after training and the retention test was performed six months after training. The main intervention was BLS education compared with BLS+WEB education. Additional training comprised self-learning training (SLT), instructor-led training (ILT), instructions from a mobile application (A), instructions from a video film (F), three reflective questions (R) and a device for feedback on compression depth (CF).

A total of 2,623 participants were cluster randomised to either BLS education or to BLS+WEB education (Fig 2 Flow-chart, www.consort-statement.org). For physical and mental reasons and due to experiencing stress and a shortage of time, 94 participants were excluded. The included participants available for analysis totalled 2,529 and their characteristics are shown in Table 1. For technical and logistical reasons, data from 104 participants were incomplete for analysis at post-test but were complete at the retention test six months later. For physical and mental reasons and due to experiencing stress and a shortage of time, 49 participants were missing at the retention test. For the primary outcome at six months, 2,480 participants were therefore analysed.

**Table 1:** Characteristics of participants.

	All participants	BLS	BLS+WEB
Variables	(n = 2529)	(n = 1293)	(n = 1236)
Age (years)	44.3±12.6	45.0±12.5	43.5±12.7%
Gender			
Male	1095 (43.3)	532 (41.1)	563 (45.6)
Female	1434 (56.7)	761 (58.9)	673 (54.4)
Body mass index (kg)	25.5±4.1	25.5±4.0	25.5±4.3
Mother tongue (1/1) ^#^			
Swedish	2182 (86.3)	1115 (86.3)	1067 (86.4)
Other	345 (13.7)	177 (13.7)	168 (13.6)
Educational level at training (1/1)			
Elementary school	226 (8.9)	129 (10.0)	97 (7.9)
High school	1230 (48.7)	660 (51.1)	570 (46.2)
College/university	1071 (42.4)	503 (38.9)	568 (46.0)
Occupation at training (1/0)			
Blue collar	1019 (40.3)	625 (48.4)	394 (31.9)
White collar	998 (39.5)	517 (40.0)	481 (38.9)
Both	511 (20.2)	150 (11.6)	361 (29.2)
Previous CPR training (3/3)			
No previous training	896 (35.5)	456 (35.3)	440 (35.7)
> 5 years ago	1627 (64.5)	834 (64.7)	793 (64.3)
Previous training on AED use (5/4)			
No previous training	2007 (79.6)	1002 (77.8)	1005 (81.6)
> 5 years ago	513 (20.4)	286 (22.2)	227 (18.4)
Previously experienced a situation of SCA (54/36)			
Yes	193 (7.9)	98 (7.9)	95 (7.9)
No	2246 (92.1)	1141 (92.1)	1105 (92.1)
Previously experienced a situation of stroke (47/50)			
Yes	287 (11.8)	155 (12.4)	132 (11.1)
No	2145 (88.2)	1091 (87.6)	1054 (88.9)
Previous experienced a situation of AMI (61/45)			
Yes	300 (12.4)	163 (13.2)	137 (11.5)
No	2123 (87.6)	1069 (86.8)	1054 (88.5)
Any known own CVD (65/59)			
Yes	157 (6.5)	82 (6.7)	75 (6.4)
No	2248 (93.5)	1146 (93.3)	1102 (93.6)
Any relative/related person with known CVD (147/138)			
Yes	936 (41.7)	499 (43.5)	437 (39.8)
No	1308 (58.3)	647 (56.5)	661 (60.2)

Data are presented as the mean±SD or n (%).

^#^Number of participants where information was missing in the two training groups respectively.

Abbreviations: BLS, basic life support education; BLS+WEB, basic life support with web-based education; CPR, cardiopulmonary resuscitation; AED, automated external defibrillation; SCA, sudden cardiac arrest; AMI, acute myocardial infarction; CVD, cardiovascular disease.

### Total score for performed practical skills in BLS

The BLS+WEB education was more effective and the participants obtained a significantly higher total score for adherence to practical skills in the BLS algorithm six months after training compared with the BLS without the web-based education (Table 2). Notably, both directly and six months after training, there was a statistically significant difference in favour of the BLS+WEB group, in addition to the BLS training. The mean value showed a visible difference, although the median value was the same in the two groups on both occasions. In contrast there was a decrease of the total score in both groups six months after training.

**Table 2:** Total score on the modified Cardiff Test for adherence to the parameters in the basic life support (BLS) algorithm.

	**BLS at post-test (%)**			**BLS at retention test (%)**		
	**BLS**	**BLS+WEB**		**BLS**	**BLS+WEB**	
**Variables**	**(n = 1213)**	**(n = 1212)**	**p-value**	**(n = 1268)**	**(n = 1212)**	**p-value**
Individual scores (min 1 point–max 6 points)						
Checks responsiveness—by talking						
2 p. Yes	97.0	97.1	0.95	94.3	96.8	0.02
1 p. No	3.0	2.9		5.7	3.2	
Checks responsiveness—by shaking						
3 p. Yes	95.4	96.9	0.16	92.5	95.8	0.005
2 p. No	4.3	3.1		6.9	4.2	
1 p. Potentially dangerous	0.3	0.1		0.6	0	
Opens airway—by head tilt and chin lift						
5 p. Perfect as instructed	51.9	54.4	0.52	34.5	40.6	0.05
4 p. Acceptable	6.0	6.6		6.5	6.7	
3 p. Attempted other	1.0	0.7		1.2	0.7	
2 p. Attempted visible, fails	15.7	17.3		23.5	23.8	
1 p. No	25.5	21.0		34.3	28.2	
Checks breathing—by look, listen and feel						
2 p. Yes	90.3	89.5	0.49	81.9	81.4	0.26
1 p. No	9.7	10.5		18.1	18.6	
Calls 112 or shouts to call 112						
2 p. Yes	94.2	95.1	0.69	93.8	96.5	0.02
1 p. No	5.8	4.9		6.2	3.5	
Asks for AED						
2 p. Yes	78.3	87.6	0.0001	79.5	86.8	0.0006
1 p. No	21.7	12.4		20.5	13.2	
Starts CPR—compression/ventilation ratio						
4 p. 30:2 (28–32:2)	87.6	88.5	0.51	74.4	76.7	0.61
3 p. Another ratio	11.3	10.7		24.4	22.3	
2 p. Compressions only	1.1	0.7		1.2	1.0	
1 p. Ventilations only	0	0		0	0	
Hand placement compressions						
4 p. Correct	21.8	21.7	0.91	25.4	20.0	0.02
3 p. Other wrong	46.2	55.3		48.2	50.0	
2 p. Too low	31.9	23.0		26.4	30.0	
1 p. Not attempted	0	0		0	0	
Average compression depth						
6 p. 50–59 mm	54.4	54.9	0.75	59.2	61.1	0.51
5 p. ≥ 60 mm	12.0	14.9		9.9	11.4	
4 p. 35–49 mm	27.0	24.8		26.6	23.6	
2 p. < 35 mm	6.7	5.4		4.3	4.0	
1 p. Not attempted	0	0		0	0	
Average compression rate						
6 p. 100–120	37.3	41.7	0.23	40.6	41.7	0.96
5 p. 121–140	11.0	12.1		13.7	16.4	
4 p. 80–99	34.0	29.8		28.2	27.3	
3 p. > 140	2.1	1.9		2.2	3.2	
2 p. < 80	15.6	14.5		15.3	11.4	
1 p. Not attempted	0	0		0	0	
Total compressions counted						
6 p. 140–190	54.7	56.6	0.91	57.3	56.3	0.28
5 p. > 190	15.0	16.0		15.3	19.2	
4 p. 121–139	14.1	13.4		12.5	11.0	
3 p. 81–120	13.3	11.9		11.8	10.6	
2 p. ≤ 80	3.0	2.1		3.1	2.9	
1 p. Not attempted	0	0		0	0	
Average ventilations volume						
5 p. 500–600 ml	7.9	9.7	0.26	10.2	9.6	0.60
4 p. 1–499 ml	10.8	11.5		12.1	12.4	
3 p. > 600 ml	70.2	68.3		63.5	66.4	
2 p. 0 ml	10.0	9.7		13.0	10.6	
1 p. Not attempted	1.1	0.7		1.2	1.0	
Total ventilations counted						
5 p. 8–12	59.9	62.0	0.82	54.7	55.9	0.74
4 p. 1–7	23.1	20.3		20.3	17.8	
3 p. > 12	6.0	7.3		10.9	14.7	
2 p. 0	10.0	9.7		13.0	10.6	
1 p. Not attempted	1.1	0.7		1.2	1.0	
Total hands-off time						
4 p. ≤ 60 seconds	4.0	3.1	0.19	5.3	5.4	0.77
3 p. 61–90 seconds	55.7	59.0		61.2	63.6	
2 p. 91–135 seconds	38.6	36.2		31.8	29.9	
1p > 135 seconds	1.7	1.7		1.7	1.1	
Switches on AED						
2 p. Yes	99.4	99.7	0.55^#^	99.8	99.8	0.68^#^
1 p. No	0.6	0.3		0.2	0.2	
Attaches electrode pads						
6 p. Both pads completely in areas	87.7	93.4	0.0008	85.6	94.3	<0.0001
5 p. One in area, one crossing border area	5.0	2.8		5.4	1.7	
4 p. One in area, one outside area	3.9	1.3		5.2	1.9	
3 p. Both crossing border of area	0.4	0.3		1.0	0.3	
2p Both outside areas	2.3	1.7		2.7	1.6	
1p Not attached or not plugged into AED	0.7	0.4		0	0.2	
Checks safety, ensures nobody in contact with the victim						
2 p. Yes	59.8	65.8	0.07	47.0	51.8	0.12
1 p. No	40.2	34.2		53.0	48.2	
Delivers shock as directed by AED						
2 p. Yes	99.3	99.5	0.61^#^	99.9	99.6	0.12^#^
1 p. No	0.7	0.5		0.1	0.4	
Resumes CPR immediately after shock						
2 p. Yes	91.6	93.6	0.04	88.8	87.7	0.34
1 p. No	8.4	6.4		11.2	12.3	
	**BLS at post-test**			**BLS at retention test**		
**Total score (19–70 points)**	**BLS**	**BLS+WEB**	**p-value**	**BLS**	**BLS+WEB**	**p-value**
Mean	58.7	59.6	0.004	58.0	58.8	0.03
Standard deviation	±4.9	±4.8		±5.0	±5.0	
Median	60	60		59.00	59.00	
25^th^,75^th^ percentile	56,62	57,63		55,62	56,62	
Min, max	35,68	37,69		41,69	37,69	

The data include results from all available data. All education groups are presented as numbers and proportions (per cent) or as means with standard deviation (SD) and medians with 25^th^, 75^th^ percentiles. The significance level was 0.05 (two-sided test). To account for a potential cluster effect of the training groups, mixed linear regression models were applied for comparisons of the total score and other continuous measurements. For comparisons of proportions, generalized estimation equations (GEE) analysis with logit link function was applied. All comparisons between the training groups were adjusted for the possible confounding influence of gender, educational level, previous CPR training and additional interventions.

^#^Fisher’s exact test was used, without adjustment for clustering or for covariates.

Abbreviations: Total score, total score of 19 points as minimum achieved and 70 points as maximum achieved for Cardiff Test; Cardiff Test, Cardiff Test for basic life support and automated external defibrillation; Individual score, individual scores from the Cardiff Test with one point as minimum achieved and six points as maximum achieved for each parameter; BLS, basic life support and automated external defibrillation; CPR-AED, cardiopulmonary resuscitation and automated external defibrillation; Post-test, practical test directly or within one day after the intervention; Retention test, practical test six months after the intervention; BLS, BLS without web-based education in addition to CPR-AED training; BLS+WEB, a preparatory web-based education in addition to the CPR-AED training.

### Separate variables for quality of performed CPR-AED

Check responsiveness, call 112, open airway, ask for an AED and attach electrode pads correctly in areas were performed significantly more effectively in the BLS+WEB group than in the BLS group, six months after training. In contrast, hand placement for compressions was performed more correctly in the BLS group six months after training, but, for average compression depth and rate, there were no significant differences between the two groups. Average ventilation volume did not show any difference but was too large in both groups.

The overall quality of compressions obtained a significantly higher score directly after training in the BLS+WEB group, but there was no difference at the six-month follow-up (Table 3). Correct compressions increased from post-test to retention test in the BLS group and decreased in the BLS+WEB group. Time to shock was shorter in the BLS+WEB group, with a significant difference six months after training.

**Table 3:** Separate variables for quality of practical skills for cardiopulmonary resuscitation and automated external defibrillation (CPR-AED).

	CPR-AED			CPR-AED		
	Post-test			Retention test		
Variables	BLS	BLS+WEB	p-value	BLS	BLS+WEB	p-value
Time to start of CPR (seconds)	N = 1213	N = 1212		N = 1268	N = 1212	
Median	28	27	0.73	27	27	0.95
25th,75th percentile	21,37	22,36		21,35	20,35	
Average compression depth (mm)	N = 1213	N = 1211		N = 1268	N = 1212	
Median	54	55	0.04	54	55	0.29
25th,75th percentile	46,58	48,59		47,58	48,58	
Average compression rate (per minute)	N = 1213	N = 1211		N = 1268	N = 1212	
Median	100	102	0.39	102	106	0.007
25th,75th percentile	86,113	88,114		87,115	91,117	
Compressions with insufficient depth (%)	N = 1205	N = 1205		N = 1268	N = 1212	
Median	11	8	0.03	11	8	0.17
25th,75th percentile	1,70	1,58		2,64	1,53	
Compressions with incorrect hand position (%)	N = 1205	N = 1205		N = 1268	N = 1212	
Median	21	20	0.20	22	26	0.22
25th,75th percentile	1,62	1,57		1,67	2,73	
Compressions with incomplete release (%)	N = 1205	N = 1205		N = 1268	N = 1212	
Median	0	0	0.61	0	0	0.18
25th,75th percentile	0,1	0,1		0,0	0,0	
>0 (%)	26.2	26.3	0.48	20.4	23.7	0.009
Correct compressions (%)	N = 1205	N = 1205		N = 1268	N = 1212	
Median	37	43	0.03	39	39	0.87
25th,75th percentile	5,79	9,80		6,78	6,78	
Average ventilation volume (ml)	N = 1213	N = 1211		N = 1268	N = 1212	
Median	873	858	0.67	735	802	0.09
25th,75th percentile	535,1244	534,1238		484,1140	507,1160	
Correct ventilations (%)	N = 1207	N = 1205		N = 1268	N = 1212	
Median	0	1	0.39	1	1	0.23
25th,75th percentile	0,2	0,2		0,3	0,3	
>0 (%)	49.8	51.7	0.35	53.8	53.1	0.32
Time to first shock (seconds)	N = 1206	N = 1209		N = 1267	N = 1210	
Median	68	65	0.21	68	65	0.0004
25th,75th percentile	59,79	56,78		59,81	57,76	

Data collected from the Resusci Anne manikin and the PC SkillReporting System (Laerdal Medical, Stavanger, Norway). All available data were used. Mixed linear regression was used, except when the distribution was too skewed. In the latter cases, the comparisons were performed on the dichotomised variable 0/>0, using generalized estimation equations (GEE) analysis. All tests are two-sided and p-values below 0.05 were considered statistically significant.

Abbreviations: CPR, cardiopulmonary resuscitation; AED, automated external defibrillation (CPR-AED); BLS, basic life support education; BLS+WEB, BLS plus web-based education.

### Self-assessed theoretical knowledge, confidence and willingness to act

The assessment of the participants theoretical knowledge of stroke and AMI symptoms and healthy lifestyle factors showed a significant difference, with a higher total score in the BLS+WEB group both directly and six months after training when compared with the BLS group (Table 4 and S1 Table). Self-assessed theoretical knowledge of CPR, AED and confidence and willingness to act in an OHCA situation did not differ between the groups directly after training or six months later.

**Table 4:** Theoretical knowledge of cardiovascular disease (CVD), self-assessed theoretical knowledge, confidence and willingness to act in a real-life OHCA situation.

	Post-test			Retention test		
	BLS	BLS+WEB		BLS	BLS+WEB	
Variables	(n = 1213)	(n = 1212)	p-value	(n = 1268)	(n = 1212)	p-value
**Total score for correct answers**						
Theoretical knowledge of first action if stroke or AMI, i.e. 112 (%) (8/4/8/3) ^#^	98.7	99.1	0.44	98.6	99.3	0.11
Theoretical knowledge of first action if OHCA, i.e. alarm 112 (%) (10/13/13/8)	77.1	88.7	<0.0001	66.5	74.7	0.001
Theoretical knowledge of stroke (mean, SD) (52/43/37/28)	4.2±2.1	5.5±1.6	<0.0001	4.6±2.0	5.7±1.5	<0.0001
Theoretical knowledge of AMI (mean, SD) (31/33/37/24)	4.5±2.4	6.4±2.1	<0.0001	4.8±2.3	6.7±2.2	<0.0001
Theoretical knowledge of healthy lifestyle factors (mean, SD) (22/9/42/24)	5.2±1.3	5.8±0.6	<0.0001	5.3±1.3	5.7±0.7	<0.0001
**Individual score for answered yes**						
Self-assessed theoretical knowledge and practical skills to be able to perform compressions (%) (135/131/195/162)	96.7	96.1	0.12	97.1	98.1	0.33
Self-assessed theoretical knowledge and practical skills to be able to perform ventilations (%) (140/126/196/168)	96.6	96.5	0.34	97.1	97.8	0.66
Self-assessed theoretical knowledge and practical skills to be able to use an AED (%) (220/172/247/190)	91.4	91.4	0.55	95.1	95.4	0.91
Self-assessed confidence after training (%) (80/72/86/65)	97.7	97.9	0.34	98.1	98.7	0.43
Self-assessed willingness to act if a relative suffers an OHCA (%) (1/0/6/1)						
Would not dare or want to intervene	1.6	0.9	0.20^##^	1.1	0.3	0.03^##^
Would give ventilations only	0.1	0.2	0.62^##^	0.6	0.2	0.34^##^
Would give chest impressions only	2.9	2.1	0.32	2.4	2.3	0.88
Would give both chest compressions and ventilations	95.5	96.8	0.21	96.0	97.1	0.22
Self-assessed willingness to act if an unknown person suffers an OHCA (%) (8/6/13/6)						
Would not dare or want to intervene	3.9	2.7	0.15	3.5	2.2	0.18
Would give ventilations only	0.3	0.2	0.73^##^	0.3	0.2	1.00^##^
Would give chest impressions only	27.3	29.1	0.18	32.7	31.9	0.96
Would give both chest compressions and ventilations	68.5	67.9	0.38	63.5	65.6	0.66

Data collected from questionnaires directly after basic life support (BLS) training (post-test) och six months after BLS training (retention test). All available data were used. All education groups were analysed. The data are presented as proportions (percent) or as crude means with standard deviation (SD).

Abbreviations: CVD, cardiovascular disease; OHCA, out-of-hospital cardiac arrest; AMI, acute myocardial infarction; 112, emergency number; AED, automated external defibrillator.

^#^Number of participants where information was missing in the two training groups at the post-test and retention test respectively.

^##^Fisher’s exact test used, without adjustment for clustering or for covariates.

## Discussion

The main findings support earlier studies emphasising a blended learning approach for education in BLS [6, 12, 17, 32]. This study found that a web-based education on CVD, in addition to training in BLS, statistically improved the participants’ learning outcome in terms of practical skills. However, since the differences were very small, the clinical relevance of our findings could be the subject of debate. The results showed that the BLS training with a preparing web-based education on CVD has benefits but with a limited practical impact.

Previous studies have reported improved theoretical knowledge but with no significant difference for the overall total score for practical skills when compared with blended learning including both digital material and an instructor present with traditional instructor-led learning [11, 13, 14, 16, 33]. With the Cardiff Test (19–70 points), we calculated the CPR quality in relation to optimal CPR ((individual total score-19)/maximum total score-19) x100), a total score of 80% directly after intervention and 78% six months after the intervention in both groups. This corresponds to Hsieh et al. [34] who reported an overall score of 79%, six months after intervention for university students. Other previous BLS studies reported results below 75% of the total score for BLS [16, 28, 29, 35], but there is no study precisely comparable to our study with a study population of laymen from workplaces. However, the total score for the Cardiff Test for BLS included several variables in the algorithm, although the early initiation of high-quality CPR is the most critical factor [2–4].

The early initiation of high-quality CPR-AED is associated with increased survival from OHCA [3, 4]. This trial showed a rapid start to CPR in both groups. The BLS+WEB group called 112 significantly more often compared with the BLS group, six months after intervention. We felt that a figure of at least 75% of the total score met the requirements for clinically relevant high-quality CPR-AED [2, 6, 16]. In this trial, we found a figure of below 43% for correct compressions for separate variables. The BLS+WEB education did not increase practical skills in terms of compressions. Furthermore, the BLS training without the web-based education resulted in an even lower score. In addition, compressions with the incorrect hand position were performed more frequently in the BLS+WEB group. Causes for the low quality may be the design of the training, the small training manikin, the web-based education or that the test was performed on a full body manikin. A comparable trial in 2018 reported 42% correct compressions three months after training for first-year medical students [36]. In comparison, other studies have reported a result of more than 75% correct compressions [17, 34] and that may be equivalent to clinical requirements. Interestingly in our study, correct compressions increased from post-test to retention test in the BLS group and decreased in the BLS+WEB group.

Other findings favouring the BLS+WEB group were that the first shock was delivered more rapidly six months after intervention and that the participants asked for the AED and attached the electrode pads completely in appropriate areas more often compared with the BLS group.

Moreover, a theoretical knowledge off calling 112 in the event of OHCA was significantly higher in the BLS+WEB group both directly and six months after intervention, but awareness was low in both groups. Theoretical knowledge of symptoms for CVD and AMI and healthy lifestyle factors was significantly higher in the BLS+WEB group. This awareness was constant after six months and in line with other studies [9–11]. Self-assessed theoretical knowledge, confidence and willingness did not differ between the groups, except for hesitation about starting CPR on a relative, which was higher in the group without the web-based education. This can be interpreted as that the web-based education needs to be improved. Further analysis of differences in willingness between the groups depending on age and gender should be considered. In a recent study with a telephone survey, Krammel et al. showed that there was a lower willingness to perform BLS and use an AED in an OHCA by elderly and women [37].

The clinical relevance of our findings could be discussed due to minor differences between the two groups in terms of the primary outcome. However, a number of secondary endpoints indicated favourable effects for the combined BLS+WEB education as compared to BLS alone. Furthermore, this combination of learning activities appears to increase public awareness of CVD, including the importance of risk factors and how to recognise stroke and AMI.

This study may be considered for further web-based education in addition to international science and practice and new perspectives [1, 32, 38–40]. E-learning prior to the advanced life support (ALS) course for medical professionals resulted in equal overall scores for practical skills when compared with traditional training in ALS. However, the e-learning reduced the total time of the education and the number of instructors and generated economic benefits [13–15]. Technological learning activities in BLS for laymen may also be cost effective [41] and may match the workplace structure for learning [39]. Practical skills in BLS decay after three to six months and, for this reason, easily available digital education courses could be beneficial, simplifying the education and matching different learning styles [6]. The time for frequent practical training [6, 34, 42] can be reduced and the training may be more effective and focus on the participants. According to Kolb’s learning theory [18], students are able to regulate their own knowledge and understanding before the practical training, in advance of achieving the appropriate skills and competence. The web-based education could be improved by encouraging the participant to reflect on their own learning in BLS and to make sense of the understanding in an abstract conceptualisation. This large RCT expands our knowledge of the participants’ learning in BLS. Although the differences between the groups were minor, we believe that an improved web-based education on CVD and treatments before BLS training for the public may be an appropriate alternative.

### Limitations

Even though the analyses were adjusted for potential cluster effects and possible confounders and accounted for additional interventions, the study design was a limiting factor. We were unable to exclude participant bias, because the participants were aware of the educational design without any exact knowledge of the different types of training alternative or the evaluation of outcome. The practical test used a full-body manikin and the training was undertaken on a small manikin and this could have affected the results. Moreover, the web-based education itself could be a limiting factor depending on its content and technology. For this study collected data answered the research question and the instruments have been used in previous studies, but this is only one way to come closer to the research problem. The validated instrument Cardiff Test was used to calculate total score for adherence to the BLS algorithm and might be a weakness depending on a large variety of variables in the test. Even if the instrument was modified to current guidelines it might not reflect a realistic cardiac arrest situation. For example, chest compressions are today highlighted and therefore we have presented separate variables in a table. The questionnaire contained some questions used in previous studies but have not been validated and this is a limiting factor. The small reported statistical difference has a limited clinical relevance and can be partly explained by a large sample size. Owing to this the applicability of the trial findings may be interpreted as low.

### Strengths

Strengths of the study was the large sample size and the controlled randomisation with lay participants from workplaces outside hospitals. This study was a pragmatic trial with an intervention carried out during practical work in a real workplace environment where education in BLS is recommended which enhances the generalisability of the results in the study. Practical training on commercial manikins and training AEDs was equal and included for all participants. The assessment was carried out on equal terms with questionnaires and a simulated scenario in a mobile and converted motorhome stationed near the workplace.

## Conclusion

A web-based education in CVD in addition to BLS training enhanced the learning outcome with a statistically significant higher total score for performed practical skills in BLS as compared to BLS training alone. However, in terms of the outcomes, the differences were minor and the clinical relevance of our findings has a limited practical impact.

## Supporting information

S1 Checklist The CONSORT 2010 statement checklist for cluster randomised trials. (DOCX)

S1 Text Information for research participants and consent form. (DOCX)

S2 Text Questionnaire, directly and six months after intervention. (DOCX)

S3 Text Modified Cardiff Test of basic life support and external defibrillation. (DOCX)

S1 Table Data from questionnaire on theoretical knowledge of symptoms of stroke and AMI and healthy lifestyle factors, directly and six months after intervention. (DOCX)
