# Supplementary material for: Effectiveness of web-based education in addition to basic life support learning activities: A cluster randomised controlled trial
Source: PLoS One. 2019 Jul 11;14(7):e0219341. doi: 10.1371/journal.pone.0219341 (PMC6622500; doi:10.1371/journal.pone.0219341)
Supplement: S1 Text — (DOCX) [file pone.0219341.s002.docx]

***Supporting information 1 (S1)***

**PARTICIPANT RESEARCH INFORMATION**

Information for participants, the research person in the study, on pedagogic aspects of education in basic life support (BLS), cardiopulmonary resuscitation and automated external defibrillation, for workplaces in Sweden, 2014-2016.

**Background**

The early start of cardiopulmonary resuscitation (CPR) with automated external defibrillation (AED) may double or quadruple survival from sudden cardiac arrest. Today, just over three million people have participated in education in CPR with AED in Sweden. Education adapted to different audiences and workplaces requires constant development to reach more participants. In this research, existing national training methods are evaluated and new ones are investigated to improve training opportunities for the public, the lay people in society. The current study is a so-called educational intervention and it is based on the national guidelines from the Swedish Resuscitation Council and guidelines from the European Resuscitation Council.

**Aim**

The aim of this study is to identify factors that may affect knowledge acquisition and retention after training in basic life support, cardiopulmonary resuscitation with automated external defibrillation.

**Request for participation**

We would like to ask you if you wish to participate in this scientific study, evaluating different training methods of cardiopulmonary resuscitation with automated external defibrillation. You will be asked to sign a separate consent form before participating.

**Implementation**

In this research study, participants at different workplaces will be trained in basic life support, cardiopulmonary resuscitation and using an automated external defibrillator. The study plans to include approximately 2,500 participants. The research explores the training method that is most effective for cardiopulmonary resuscitation with automated external defibrillation. Questionnaires will be handed out and the quality of cardiopulmonary resuscitation with automated external defibrillation will be measured. This means that we would like to meet all the participants for a follow-up after training and again after six months. We also intend to contact you during a five- to ten-year period for further follow-up.

**Research data**

Personal data, data collection and the processing of results will be confidential and in accordance with the Personal Data Act. Personal data will be replaced by a code. The research data contain data on parameters relating to cardiopulmonary resuscitation with automated external defibrillation, ID, age, weight, gender and workplace. The questionnaires contain questions on personal characteristics, knowledge of cardiovascular disease and cardiopulmonary resuscitation with automated external defibrillation and questions on the education. We aim to protect personal integrity. The follow-up and evaluation will take place in a single room. Interviews will be transcribed, and videotapes and results will be processed and they will be secured from unauthorised persons. When publishing research results, only data will be published without being linked to the research person. The data will be stored in a database at the Register Centre Västra Götaland in Gothenburg. Data with data relative to each other will be stored in two different places. The data will be stored for at least 10 years to enable controls. Every workplace and research person should receive written and oral information and be given an opportunity for prior approval.

**Voluntary**Participation in the research is voluntary and can be cancelled at any time. To participate as a research person, the inclusion criteria are adults, over eighteen years of age, working at the workplace in the project, never or no training in CPR-AED within the past five years. For information, please contact the contact person or the principal investigator.

**Consent form**A personal signed consent form is required from each research person before participating.

**Contact information**

Principal investigator

Johan Herlitz

Professor

Forum for Prehospital Research and Development, FOU in the Västra Götaland Region

University of Borås

SE-501 90 Borås

Sweden

+46 33 435 40 00

johan.herlitz@hb.se

Responsible for the study and contact person

Helene Bylow

University of Gothenburg

Box 100

SE-405 30 Gothenburg

Sweden

Helene Bylow

Skansasjövägen 19

SE-516 96 Aplared

Sweden

+46 705 28 69 28

[kontakt@helenebylow.se](mailto:kontakt@helenebylow.se)

helene.bylow@gu.se

***Supporting information 1 (S1)***

CODE NUMBER

**CONSENT FORM**

**in addition to the research person information**

APPROVAL OF AND CONSENT FOR THE RESEARCH:

Pedagogic aspects of education in basic life support

Before signing this consent form, please read the research person information that you have received.

According to the law (2003:460) on the ethical testing of research relating to humans, we wish to inform you, as a research person, that participation is voluntary and can be terminated at any time. You should have both received oral information and read the written research information. The research contents; training in basic life support, cardiopulmonary resuscitation with automated external defibrillation, questionnaires, personal follow-up after the training and six months after the training. We also wish to be able to contact you again in a five- to ten-year period.

For any questions on the research please contact:

Helene Bylow

Mobile: +46 705 28 69 28

E-mail: kontakt@helenebylow.se

**I hereby agree to participate and be a research person in the research project on education and training in BLS, cardiopulmonary resuscitation and automated external defibrillation.**

Date:

Workplace:

E-mail:

Mobile number:

Personal identity number:

Signature:

Print name:
