# Supplementary material for: Effectiveness of web-based education in addition to basic life support learning activities: A cluster randomised controlled trial
Source: PLoS One. 2019 Jul 11;14(7):e0219341. doi: 10.1371/journal.pone.0219341 (PMC6622500; doi:10.1371/journal.pone.0219341)
Supplement: S1 Table — (DOCX) [file pone.0219341.s005.docx]

**Supporting information 1 Table (S1 Table). Table 5. Assessment of theoretical knowledge of stroke, acute myocardial infarction and healthy lifestyle factors**

Theoretical knowledge of stroke

Post-test (%) Retention test (%)

BLS BLS+WEB BLS BLS+WEB

Variables (n=1213) (n=1212) p-value (n=1268) (n=1212) p-value

Pain on one side of the body (9/11/8/5) ^#^

Yes 40.3 37.7 0.19 43.3 38.9 0.03

**No 29.8 50.2 <0.0001 34.8 52.1** <0.0001

Do not know 29.9 12.1 <0.0001 21.8 9.0 <0.0001

Pain on both the left and the right

side of the body (17/17/18/13)

Yes 4.3 5.6 0.19 5.5 4.3 0.36

**No 58.5 80.5** <0.0001 **67.1 84.9** <0.0001

Do not know 37.2 13.9 <0.0001 27.4 10.8 <0.0001

Weakness on one side of the body (5/1/2/0)

**Yes 78.6 93.5** <0.0001 **82.1 95.1** <0.0001

No 2.3 2.3 0.73 3.7 1.8 0.02

Do not know 19.1 4.2 <0.0001 14.2 3.1 <0.0001

Weakness on both the left and the right

side of the body (20/19/21/13)

Yes 11.1 13.8 0.04 13.2 9.8 0.02

**No 53.1 70.8** <0.0001 **60.9 78.3** <0.0001

Do not know 35.7 15.3 <0.0001 25.8 11.8 <0.0001

Symptoms occur slowly (18/16/13/10)

Yes 15.6 13.0 0.07 17.2 13.1 0.002

**No 48.1 72.7** <0.0001 **52.9 75.3** <0.0001

Do not know 36.2 14.3 <0.0001 29.9 11.6 <0.0001

Symptoms occur quickly (5/3/8/0)

**Yes 64.7 86.6** <0.0001 **68.7 87.2** <0.0001

No 6.8 4.1 0.005 8.8 5.8 0.007

Do not know 28.6 9.3 <0.0001 22.5 7.0 <0.0001

Difficulty speaking or slurred speech (0/0/2/0)

**Yes 92.6 98.6** <0.0001 **93.7 99.2** <0.0001

No 0.3 0.1 0.37^##^ 0.1 0.2 0.62^##^

Do not know 7.1 1.3 <0.0001 6.2 0.7 <0.0001

Total score (52/43/37/28) 4.2±2.1 5.5±1.6 <0.0001 4.6±2.0 5.7±1.5 <0.0001

Theoretical knowledge of acute myocardial infarction

Post-test (%) Retention test (%)

BLS BLS+WEB BLS BLS+WEB

Variables (n=1213) (n=1212) p-value (n=1268) (n=1212) p-value

Discomfort or pain in the right arm (19/22/22/7) ^#^

**Yes 25.1 53.4** <0.0001 **25.6 53.3** <0.0001

No 47.2 35.1 <0.0001 50.5 35.9 <0.0001

Do not know 27.6 11.4 <0.0001 23.9 10.8 <0.0001

Discomfort or pain in the left arm (1/2/4/1)

**Yes 74.9 88.2** <0.0001 **75.2 87.9** <0.0001

No 6.4 5.5 0.33 7.8 6.1 0.04

Do not know 18.7 6.3 <0.0001 17.0 5.9 <0.0001

Discomfort or pain in the chest (2/2/3/2)

**Yes 90.8 98.8** <0.0001 **93.1 98.8** <0.0001

No 0.2 0.1 1.00^##^ 0.6 0.1 0.04^##^

Do not know 9.0 1.2 <0.0001 6.2 1.2 <0.0001

Discomfort or pain in the right leg (4/5/7/6)

Yes 2.2 3.5 0.05 3.5 2.7 0.20

**No 56.0 80.9** <0.0001 **60.3 83.3** <0.0001

Do not know 41.8 15.6 <0.0001 36.2 14.0 <0.0001

Discomfort or pain in the left leg (5/4/5/5)

Yes 6.9 6.8 0.92 8.2 5.7 0.05

**No 50.6 76.7** <0.0001 **55.3 80.0** <0.0001

Do not know 42.5 16.6 <0.0001 36.6 14.3 <0.0001

Discomfort or pain in the back (6/3/8/5)

**Yes 38.8 57.0** <0.0001 **40.0 66.4** <0.0001

No 17.8 24.7 <0.0001 20.7 19.6 0.93

Do not know 43.4 18.3 <0.0001 39.3 14.1 <0.0001

Discomfort or pain in the stomach (9/4/6/6)

**Yes 31.6 58.2** <0.0001 **36.1 65.4** <0.0001

No 24.0 24.7 0.49 25.5 19.8 0.01

Do not know 44.4 17.1 <0.0001 38.4 14.8 <0.0001

Headache (6/8/14/9)

Yes 24.0 20.3 0.02 28.5 23.7 0.007

**No 25.2 53.2** <0.0001 **29.7 55.2** <0.0001

Do not know 50.8 26.5 <0.0001 41.7 21.1 <0.0001

Nausea (4/2/8/5)

**Yes 59.6 72.7** <0.0001 **62.9 79.5** <0.0001

No 7.9 11.2 0.0004 8.7 8.1 0.78

Do not know 32.5 16.0 <0.0001 28.3 12.3 <0.0001

Total score (31/33/37/24) 4.5±2.4 6.4±2.1 <0.0001 4.8±2.3 6.7±2.2 <0.0001

Theoretical knowledge of healthy lifestyle factors

Post-test Retention test

BLS BLS+WEB BLS BLS+WEB

Variables (n=1213) (n=1212) p-value (n=1268) (n=1212) p-value

Regular physical exercise (1/2/2/0) ^#^

**Yes 91.4 98.8** <0.0001 **95.0 98.8** <0.0001

No 2.7 0.3 0.0006^##^ 0.9 0.4 0.21^##^

Do not know 5.9 0.9 <0.0001 4.1 0.8 0.0001

Smoking (9/5/18/8)

Yes 0.3 1.0 0.09^##^ 0.7 0.9 0.66^##^

**No 94.3 98.5** <0.0001 **94.7 98.7** <0.0001

Do not know 5.4 0.5 <0.0001 4.6 0.4 <0.0001

Eating fruit and vegetables daily (3/2/9/6)

**Yes 82.3 95.4** <0.0001 **81.8 93.0** <0.0001

No 1.8 1.3 0.42 3.3 2.0 0.07

Do not know 15.9 3.3 <0.0001 14.9 5.0 <0.0001

Being mostly sedentary daily (11/4/31/10)

Yes 0.4 0.2 0.45^##^ 0.6 0.7 1.00^##^

**No 91.3 98.8** <0.0001 **92.1 97.9** <0.0001

Do not know 8.2 1.0 <0.0001 7.3 1.4 <0.0001

Eating fish two or three times a week (10/6/17/12)

**Yes 64.5 89.3** <0.0001 **68.5 86.1** <0.0001

No 4.7 2.0 0.0004 7.5 4.2 0.002

Do not know 30.8 8.7 <0.0001 24.0 9.7 <0.0001

Daily exercise such as walking or cycling (2/3/5/2)

**Yes 96.4 99.5** <0.0001 **96.3 99.3**  0.0003

No 0.0 0.1 0.49^##^ 0.3 0.4 0.75^##^

Do not know 3.6 0.4 <0.0001 3.4 0.3 <0.0001

Total score (22/9/42/24) 5.2±1.3 5.8±0.6 <0.0001 5.3±1.3 5.7±0.7 <0.0001

Data collected from questionnaires answered directly after basic life support (BLS) training (post-test) and six months after BLS training (retention test). The BLS education group was compared with the BLS plus web-based education (BLS+WEB) group.

^#^ Number of participants where information was missing in the two training groups at the post-test and retention test respectively

^##^ Fisher’s exact test used, without adjustment for clustering or for covariates
